# The Long Way to Establish the Ergogenic Effect of Caffeine on Strength Performance: An Overview Review

**DOI:** 10.3390/nu15051178

**Published:** 2023-02-27

**Authors:** Verónica Giráldez-Costas, Juan Del Coso, Asier Mañas, Juan José Salinero

**Affiliations:** 1 Exercise Physiology Laboratory, Camilo José Cela University, 28692 Madrid, Spain; 2 Strength Training & Neuromuscular Performance (STreNgthP) Research Group, Camilo José Cela University, 28692 Madrid, Spain; 3 Centre for Sport Studies, Rey Juan Carlos University, 28942 Fuenlabrada, Spain; 4 GENUD Toledo Research Group, University of Castilla-La Mancha, 45071 Toledo, Spain; 5 CIBER of Frailty and Healthy Aging (CIBERFES), Instituto de Salud Carlos III, 28029 Madrid, Spain; 6 Center UCM-ISCIII for Human Evolution and Behavior, 28029 Madrid, Spain; 7 Faculty of Education, Complutense University of Madrid, 28040 Madrid, Spain; 8 Sport Training Laboratory, Faculty of Sport Sciences, Castilla-La Mancha University, 45071 Toledo, Spain

**Keywords:** coffee, ergogenic aid, muscle force, muscle power, stimulant, exercise performance

## Abstract

This overview review aimed to describe the evolution of the characteristics of the research on caffeine effects on strength. A total of 189 experimental studies with 3459 participants were included. The median sample size was 15 participants, with an over-representation of men vs. women (79.4 vs. 20.6%). Studies on young participants and elders were scarce (4.2%). Most studies tested a single dose of caffeine (87.3%), while 72.0% used doses adjusted to body mass. Single-dose studies ranged from 1.7 to 7 mg/kg (4.8 ± 1.4 mg/kg), while dose–response studies ranged from 1 to 12 mg/kg. Caffeine was mixed with other substances in 27.0% of studies, although only 10.1% of studies analyzed the caffeine interaction with these substances. Capsules (51.9%) and beverages (41.3%) were the most common forms of caffeine administration. Similar proportions of studies focused on upper (24.9%) or lower body strength 37.6% (37.6% both). Participants’ daily intake of caffeine was reported in 68.3% of studies. Overall, the pattern in the study of caffeine’s effects on strength performance has been carried out with experiments including 11–15 adults, using a single and moderate dose of caffeine adjusted to participants’ body mass in the form of a capsule.

## 1. Introduction

While the first reviews that summarized findings of studies on caffeine and sports performance confirmed the ergogenic effect of caffeine on aerobic performance already in the 1980s [1,2], the potential benefit of caffeine supplementation on muscle strength was rejected for several years [1,2,3]. In the 1980s, the paucity of studies, especially in vivo, the small sample sizes used in most of the studies, and the different types and doses of caffeine doses used made it difficult to establish solid conclusions, as the benefits of caffeine to enhance muscle strength were present in some but not all studies [1,2,3]. At that time, most of the studies tested caffeine properties in vitro using animal muscle tissues [1,2,4,5,6,7]. These in vitro studies reported the effects of caffeine on muscle performance including enhanced contractile status, potentiation of the rate of substrate utilization, facilitation of neuromuscular transmission, and alteration of Ca^2+^ permeability and release in the sarcoplasmic reticulum [1,2]. Nevertheless, empirical studies in vivo in humans were scarce and so, it was difficult to translate the benefits of animal research to human performance. As a result, systematic reviews argued that it was possible that the caffeine-induced increases in muscle contractility seen in vitro did not translate into improved strength in vivo [3].

However, the status of caffeine as a substance with a potential ergogenic effect for strength performance started to change at the beginning of the new century. In the first decade of the 21st century, several systematic reviews pointed to several benefits of acute caffeine intake on anaerobic performance, including strength, although authors still suggested that the paucity and contradictory results of studies until that date impeded a clear categorization of caffeine as an ergogenic aid for strength performance [8,9,10]. For example, studies examining the effect of caffeine on isokinetic peak torque, isometric maximal force, muscular endurance for upper body musculature, and one-repetition maximum (1RM) showed equivocal results, with caffeine having a minimal ergogenic effect within these areas [10]. However, studies suggested that caffeine could enhance contractility through local actions in the skeletal muscle itself continuing with the view of the previous decades [8,9,10,11]. Indeed, at the end of the first decade of the century, results continued equivocal, and implications of the ergogenic potential remained unclear.

Currently, there is solid evidence supporting that acute caffeine intake (habitually from 3 to 9 mg/kg) increases maximal muscle strength, power output, and strength endurance. The categorization of caffeine as an ergogenic aid for muscle performance is based upon dozens of original studies carried out in the last years, and subsequent systematic reviews that concluded that caffeine ingestion improves 1RM, isometric and isokinetic strength, the rate of force development as well as muscular endurance, velocity, and power in different resistance exercises [12,13,14,15,16,17]. Indeed, an umbrella review of 21 published meta-analyses [18] that determined the effect of caffeine in several conditions associated to exercise showed an ergogenic effect of caffeine on muscle strength among other conditions. Not only the status of caffeine as an ergogenic aid for muscle strength has changed in the last years; the main mechanism associated with caffeine’s ergogenicity in all-out exercise situations has been shifted from local (within the muscle) to central (within the central nervous system). As indicated above, seminal studies on the effect of caffeine on muscle performance hypothesized that the ergogenic effect of caffeine could be attributable (almost in part) to peripheral factors by increased sarcoplasmic reticulum calcium ion release and increased muscle contractility [1,2]. Nowadays, it has been suggested that to obtain such an effect within the muscle with caffeine, there are needed doses of caffeine that would be toxic for humans [19]. On the other hand, the assumption that caffeine’s ergogenicity is associated with the binding capacity of caffeine to block adenosine receptors (impeding the fatiguing effect of adenosine on the central nervous system) [20,21] and the caffeine-induced increase in motor-unit recruitment [11,22] are the main hypotheses of experts in the field to explain the benefit of caffeine on exercise situations that imply maximal strength production [13].

Nowadays, caffeine is widely consumed in the sports context, irrespective of the type of sport or the fitness level of the athlete. According to the caffeine concentrations of urine samples obtained for doping analysis in national and international competitions held in Spain, three out of four elite athletes consumed this substance before or during sports competitions [23]. Although there is a trend for higher urine caffeine concentrations in sports with an aerobic nature, the presence of urine is common in all types of sports, including those where maximum strength/power is key for performance such as weightlifting and judo [23]. The wide use of caffeine as a performance-enhancing substance in sports is probably linked to the solid evidence that supports the ergogenic effect of caffeine on a spectrum of exercise situations [12,13,18,24], including aerobic [13,18] and anaerobic [13,14,15,25] performance. Nevertheless, the knowledge about caffeine’s ergogenicity in exercise and sport has come a long way to be where it is nowadays, as seminal investigations did not report the ergogenic effect of caffeine on strength performance [1,2,3]. Many potential factors could be responsible for the winding path on this topic of research. Differences in study designs, such as participants’ characteristics (age, sex, training status, habitual caffeine consumption), sample size, caffeine dose, the timing of ingestion, caffeine form of administration, or successful blinding of caffeine ingestion, among others, have likely propitiated the evolution in the evidence on caffeine as an ergogenic aid for strength performance in the last decades. From a historical perspective, analyzing evolution in these key design variables could be interesting to better comprehend the current state of the art regarding caffeine’s ergogenic benefits on strength. The purpose of this overview review was to describe the evolution of the characteristics of the research on caffeine ergogenic effects on strength, focusing on participants’ attributes, experimental designs, and caffeine dose and form of administration employed. Outcomes about the ergogenic effects of caffeine on strength performance are out of the scope of this overview review, as they had been well established in previous systematic reviews and meta-analyses [2,5,6,7,20].

## 2. Materials and Methods

### 2.1. Search Strategy

The search for published studies on the topic was conducted in the databases PubMed, Scopus and Web of Science (WoS) on 10 January 2023, and it included all research published until 31 December 2022, with no year restriction. Search terms included free-text words for key concepts related to caffeine and strength performance. The full search criteria for the PubMed database was: (caffeine[Title/Abstract] OR energy drink[Title/Abstract] OR coffee[Title/Abstract] OR caffeinated[Title/Abstract]) AND (resistance exercise[Title/Abstract] OR muscle development[Title/Abstract] OR muscle strength[Title/Abstract] OR “strength training”[Title/Abstract] OR “muscle hypertrophy”[Title/Abstract] OR “power production”[Title/Abstract] OR “maximal strength”[Title/Abstract] OR “peak power”[Title/Abstract] OR plyometric[Title/Abstract] OR “force production”[Title/Abstract] OR “resistance training”[Title/Abstract] OR MVC[Title/Abstract] OR “muscle power”[Title/Abstract] OR “maximal voluntary contraction”[Title/Abstract] OR 1RM[Title/Abstract] OR “1-repetition maximum”[Title/Abstract]). Full search criteria for Scopus and WoS can be found in Supplementary 1. The search results were downloaded to a Microsoft Excel spreadsheet (Microsoft Corporation, Redmond, WA, USA) and subsequently filtered. Titles and abstracts were then screened for a later full-text review. The search for published studies was independently performed by two authors (VGC and JJS) and disagreements were resolved through discussion. A secondary search was performed by conducting forward citation tracking of reviews and meta-analyses on caffeine and strength.

### 2.2. Inclusion and Exclusion Criteria

**Figure 1:**
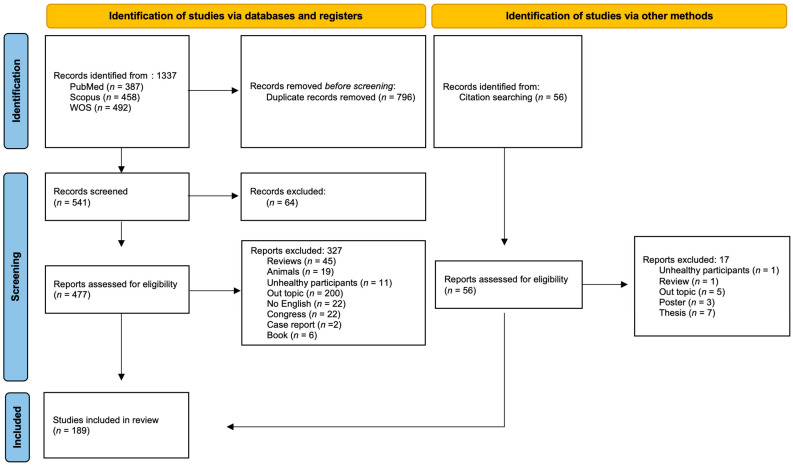
Flow diagram of the search and selection process.

To warrant inclusion in the current analysis, potential studies were required to meet the following criteria: (a) experimental trial; (b) carried out in human participants of either sex (or samples including participants of both sexes) and at all age groups; (c) used healthy participants without known chronic disease or injury; and (d) studies on the effects of oral caffeine intake on variables associated to strength performance. Systematic reviews and meta-analyses were excluded, in addition to those original studies with no full-text available, nor peer-reviewed articles, opinion pieces, commentaries, case reports, and editorials. Conference proceedings and poster presentations were also excluded, as it was unfeasible to certify the review process and to avoid duplication with original studies. Figure 1 depicts the details of the study selection methodology. After the removal of duplicates and the application of inclusion/exclusion criteria, a total of 189 studies were included in this review.

### 2.3. Data Extraction

Once the inclusion/exclusion criteria were applied, the following information was tabulated on a predefined coding spreadsheet independently by two authors (VGC and JJS) using Microsoft Excel (Supplementary 2): (a) author(s), title and year of publication; (b) sample size, participants’ sex and age; (c) caffeine form and dosage; (d) whether the experiment included an absolute (in mg) or body mass-adjusted dose of caffeine (mg/kg) and whether it was a single dose or a dose–response study; (e) whether caffeine was administered purely (e.g., caffeine anhydrous) or in a supplement or foodstuff that contained other substances (e.g., coffee, energy drinks, etc.); (f) habitual caffeine intake of the participants; and (g) reported side effects. Subsequently, disagreements were resolved through discussion until a consensus was achieved. Studies were grouped by the year of publication using the following groups: before 1980, 1980–1999, 2000–2009, 2010–2014 and 2015–2022. This grouping was created to offer a historical perspective of the studies published on the topic but increasing the sensitivity in the last years, as there has been a higher number of studies published.

### 2.4. Statistical Analyses

All the data were analyzed with the statistical package Jamovi v.2.3 [26]. Quantitative variables are reported as mean ± standard deviation (SD). Absolute and relative frequencies were calculated to describe qualitative variables. Crosstabs with chi-square statistics were calculated to analyze differences between groups in qualitative variables. The Kolmogorov–Smirnov test was used to confirm the normality of the quantitative variables. Kruskal–Wallis tests were used to analyze differences between years-groups in quantitative variables as they had no normal distribution. The significance level was set at *p* < 0.05.

## 3. Results

### 3.1. Main Search

Figure 1 depicts the flow diagram of the search and the screening process. The initial search yielded 1337 studies. After the duplicates were removed, 541 studies were entered for the title and abstract screening. Subsequently, 477 items were selected for full-text review, with 327 excluded for the following reasons: 45 were reviews, 19 were conducted in animals, 11 were included samples of participants with a known disease, 200 were out of the scope of this review (i.e., lack of variables associated to strength performance), 22 were not written in English and 30 were congress abstracts, case reports or books. In addition, 56 records were identified through the search of citations included in reviews and meta-analyses on caffeine and strength, of which 17 were excluded for the following reasons: one was carried out in a sample of participants with a known disease, one was a systematic review, five did not include measurements on strength performance variables, three were poster presentations and seven were doctoral theses. Finally, a total of 189 studies analyzing the potential ergogenic effect of caffeine on strength performance were included in this overview review.

**Figure 2:**
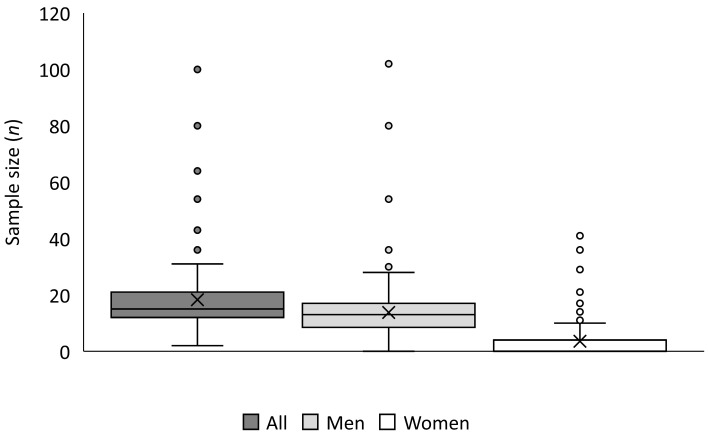
Number of papers published according to sample size and participants’ gender.

Participants. Within the studies included in the review, there was a total of 3459 participants (2606 males, 676 females, five papers did not inform about participants’ sex [27,28,29,30,31], and it was unfeasible to ascertain the gender of 177 participants). The mean sample size was 18.3 ± 13.0 participants (median = 15), with a larger number of men than women (14.2 ± 11.9 men/study vs. 3.7 ± 7.0 women/study, respectively), being the median of 13 participants for men’s studies and 0 participants for women’s studies (Figure 2). Overall, 68.3% (129) of the studies did not include any female participants, while only 12.2% (23) of the studies did not include male participants. The most frequent sample size was between 11 and 15 participants (71; 37.6%), while 85.7% of studies included between six and 25 participants. Most of the studies were performed including young adults, with mean ages between 18 and 35 years. Only five studies employed participants with a mean age lower than 18 years [30,32,33,34,35], one with middle-aged women [36], and two with older people [37,38]. Two papers did not show data on participants’ age [39,40]. According to training status, 6 (3.2%) papers described participants as untrained, 19 (10.1%) papers described participants as active, and 137 (72.5%) indicated some level of training. Three studies (1.6%) mixed trained and untrained participants [41,42,43] and 24 (12.7%) did not inform about the participants’ training status.

**Figure 3:**
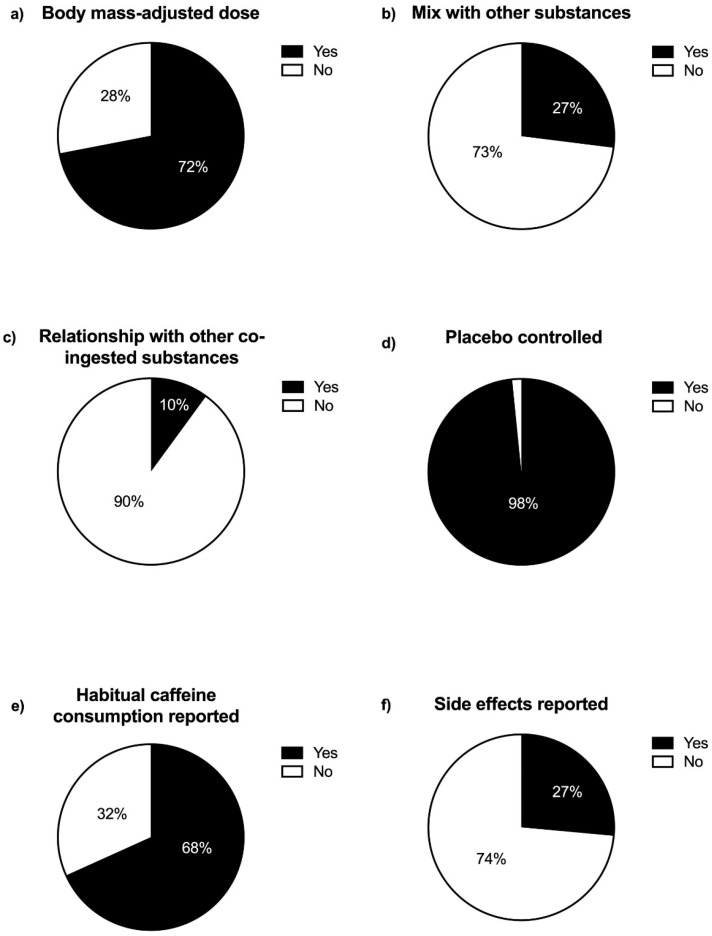
Frequency of studies published on the effect of caffeine on strength performance according to the use (or not) of a body-mass adjusted dose of caffeine (**a**), a combination of caffeine with other substances (**b**), whether a relationship with other co-ingested substances (**c**), employed a placebo-controlled situation (**d**), reported participants’ habitual caffeine consumption (**e**), and reported caffeine-associated side effects (**f**).

Caffeine supplementation. Figure 3 (panel a) depicts that 72.0% (136) of the studies used body mass-adjusted caffeine doses (unique dose or dose–response design). Studies with a body mass-adjusted and a single dose of caffeine (61.4%; 116) used a mean dose of 4.8 ± 1.4 mg/kg (range from 1.7 to 7 mg/kg). In addition, 20 (10.6%) studies compared more than one body mass-adjusted dose of caffeine in a dose–response design, including from low to high doses (ranging from 1 to 12 mg/kg). In contrast, 28.0% (53) of studies used an absolute dose of caffeine for all participants, ranging from low doses of 50 to 800 mg. In studies with a single absolute dose of caffeine, the mean value employed was 276.1 ± 134.0 mg (four studies did not show the fix-dosage used) [41,44,45,46]. Only four (2.1%) studies compared different fixed dosages, including low to high doses (from 100 to 750 mg). From the total, 73.0% (138) of studies used caffeine in a pure/isolated form (Figure 3, panel b). Only 10.1% (19) of studies analyzed the interaction of caffeine with other co-ingested substances (Figure 3, panel c). Approximately half of the studies used caffeine in capsules (51.9%, 98), while 41.3% (78) used caffeine-containing beverages.

Study design and other outcomes are measured. Almost all the analyzed studies (98.4%, 186) used a placebo-controlled design (Figure 3, panel d), and 68.3% (129) of the studies reported participants’ daily caffeine consumption (Figure 3, panel e). However, the study of the side effects produced by caffeine ingestion was registered only in 26.5% (50) of studies (Figure 3, panel f). Overall, upper and lower body strength has been similarly investigated, with 24.9% (47) papers analyzing only upper body strength, 37.6% (71) focusing on only lower body strength, and 37.6% (71) including both upper and lower body strength exercises.

### 3.2. Temporal Analysis

The first paper included in this review data was from 1907. However, research about caffeine and strength performance was scarce in the 20th century. Most of the research on this topic has been published in the 21st century (94.2%, 178). Our data showed that only three studies (1.6%) were included before 1980; eight studies (4.2%) were published between 1980 and 1999; 18 (9.5%) were published between 2000 and 2009, 29 (15.3%) were published between 2010 and 2014; and 131 studies (69.3%) were published between 2015 and 2022. Table 1 shows the temporal evolution of samples, doses, and methodological characteristics of studies related to the effects of caffeine on strength performance.

Participants. The sample size was similar among the year groups established for this review, except for <1980 with respect to 2010–2014 and 2015–2022 (*p* < 0.05) with lower sample sizes before 1980. The mean number of male participants was 2.3 ± 0.6, and there were no female participants in studies before 1980. Between 1980 and 1999, the mean number of male participants was 14.7 ± 7.0 and 2.3 ± 6.0 for female participants. Between 2000 and 2009, mean values for male and female participants were 13.8 ± 5.6 and 1.9 ± 4.4, respectively. Between 2010 and 2014, mean values for male and female participants were 13.8 ± 6.7 and 1.3 ± 3.4 participants and mean values between 2015 and 2022 were 14.6 ± 13.6 and 4.6 ± 7.8 participants, respectively.

Caffeine supplementation. Except for studies before 1980, studies employing body mass-adjusted doses were the most common (from 62.5% to 77.8%; *p* = 0.07). Studies with dose–response designs were lower in number with respect to those that used a single dose of caffeine in all the year groups included in this review. The higher proportions of dose–response designs were found in 1980–1999 (25.0%, 2) and 2015–2022 (15.3%, 20) although without significant differences with respect to the other year groups (*p* = 0.21). Studies from 1980 to 1999 used the greater mean doses in both body mass-adjusted doses of caffeine (5.8 ± 0.8 mg/kg) and fixed doses of caffeine (437.9 ± 69.0 mg). There were no significant differences in the doses of caffeine employed over time in studies with adjusted-to-body-mass doses of caffeine or studies with a fixed dose (*p* > 0.05). There were significant differences in the use of isolated vs. mixed forms of caffeine administration among the year groups (*p* < 0.01). In all year groups, except for 2010–2014, the effect of caffeine was mostly investigated in an isolated form, ranging from 66.7% to 87.5%. However, for the 2010–2014 period, only 44.8% of studies used pure caffeine administration. Overall, only a few studies (about 10%, except for studies before 2000 where there were no studies; *p* = 0.76) analyzed the potential interaction of caffeine with other substances in multi-ingredient studies. The temporal evolution of the methodological characteristics was also reflected in the form in which caffeine was ingested in the studies. Overall, the results showed that capsules or tablets were the most used form of administration (51.9%, 98); the second most used form of administration was through beverages (coffee, energy drinks, or caffeine powder dissolved in liquid, 41.3%, 78). Since 2015, other forms of caffeine administration have appeared, such as gels (0.8%, one study) [47], chewing gum (2.3%, three studies) [48,49,50], and mouth rinse (3.8%, five studies). The studies before 2000 analyzed to a greater extent the effect of caffeine on exercise that implied lower body strength, while in the remaining year groups, there was a comparable proportion of studies on both lower and upper body strength (*p* = 0.05).

Study design and other outcomes measured. The results showed that over time, almost all of the studies were placebo-controlled experiments, except in the studies before 1980 (*p* < 0.01). Before 1980, no studies reported habitual caffeine consumption or side effects. After 1980, approximately two out of three studies included information about participants’ daily caffeine consumption (*p* = 0.13), but only a minority (27.5%) reported side effects (*p* = 0.50). Until 2000, studies performed on the upper body were scarce (only three studies), but this situation changed in the subsequent years with 44 new studies (total 47; 24.9%) on caffeine’s effects on upper body strength performance and 71 studies (37.6%) on both upper and lower body strength performance (*p* = 0.05). In the last years, a similar proportion of lower and upper body studies have been performed.

## 4. Discussion

The purpose of this overview review was to describe the evolution of the characteristics of the research on caffeine ergogenic effects on strength, focusing on participants’ attributes, experimental designs, and caffeine dose and form of administration employed. The principal results of this review show an increasing interest in the effect of caffeine on strength performance in the last years, at least in the number of studies published. Additionally, most of the research on this topic used placebo-controlled experiments, which were performed in samples of 20 or fewer young adults (18 to 35 years old) with a certain level of training. Although the tendency is changing slowly in the last years, still males are feature several times more frequently than women in samples of these studies. Most of the investigations used isolated caffeine in capsules, and the dose is adjusted to the participant’s body mass (on average, ~5 mg/kg). The inclusion of measurements to report the habitual caffeine intake of participants is relatively common, especially in the last years, but the inclusion of assessments about caffeine-induced side effects during and after exercise is still included in a small proportion of studies.

Throughout history, there have been no relevant changes in the research designs employed to determine the ergogenic effect of caffeine on strength performance (except for the seminal studies) nor about the doses of caffeine used in these experiments. Therefore, we believe that the lack of evidence on the effects of caffeine on strength performance up to the beginning of the 21st century [8,9,10] was based on the scarcity of studies carried out up to that time. Reviews performed in the early 2000s argued that evidence on caffeine’s potential benefit on strength-based exercise was in its infancy but was promising [8]. Indeed, some years after, Burke [9] and Davis [10] reported that studies examining caffeine’s effect on strength outcomes showed equivocal results, with caffeine having minimal ergogenic effect on this type of exercise. Nowadays, there is solid evidence about the ergogenic effects of caffeine on strength performance. The high volume of publications in the last decade has facilitated multiple systematic reviews [15,16], which have highlighted the positive effects of caffeine on strength performance.

The use of caffeine supplementation to improve physical performance in humans is not a new field of study. Although a few investigations were carried out to demonstrate the benefits of caffeine in German laboratories toward the end of the 19th century [51], we can assume that the beginning of the experimentation on caffeine’s ergogenicity in humans was set in the early 1900s. In 1907, Rivers and Webber [39] were the first researchers interested in the effects of caffeine on muscular performance. These researchers studied the effect of caffeine on themselves, including a placebo trial and a blinding protocol (never performed before that date) to isolate the effect of caffeine on human performance over other confounding variables. This pioneering work reported an increase in the capacity for muscular work with a dose of 300 mg of caffeine citrate, but the increase was of different magnitude between the only two participants included in the study. Afterwards, Hyde and Root in 1917 [41] analyzed two healthy men, who did more than twice as much work on the ergograph when ingesting caffeine. In 1939, Thornton et al. [40] reported an increase in performance in reaction time, tapping tests, hand grip strength, and maintained hand grip with a dose of 300 mg of caffeine compared to a placebo (*n* = 3). Although these seminal works used adequate experimental designs with controls such as previous exercise, time of the day, or the use of placebo trials, few investigations of caffeine occurred before the 1980s, and sample sizes were too small to establish unequivocal conclusions. Consequently, the effect of oral intake of caffeine on muscle strength was considered as poorly studied, and the ergogenic effects of caffeine were rejected in the 1980s [1,2,3]. In addition, caffeine was catalogued as a banned substance in sports (prohibited only in competition) by anti-doping authorities between 1984 and 2004. A high threshold (12 μg/mL) for urine caffeine concentration was set in 1987 to limit the use of high doses of caffeine, and only athletes that surpassed this threshold were penalized for doping misconduct. Maybe, the consideration of caffeine as a doping agent led to lower research about caffeine and strength performance. In the two decades that caffeine was considered a banned substance, only nine studies were published on this topic. In 2004, the World Anti-Doping Agency decided to remove caffeine from the list of banned substances, and since then, athletes have been able to consume caffeine in any form freely. However, the use of caffeine in athletes of strength-based sports disciplines just after the removal of caffeine from the list of banned substances was low [52], as there was no evidence to support its use. Interestingly, in the first decade after the removal of caffeine from the banned list (2005–2015), 58 studies were conducted on the effect of caffeine on strength performance. This increase in the research interest regarding the benefits of caffeine on strength was accompanied by increases in the use of caffeine in sports such as weightlifting, judo, and boxing, at least judged by the post-competition urinary concentrations of athletes of these disciplines between 2004 and 2015 [23]. Perhaps, the fact that the effects of caffeine continued to be contradictory even with the rise in research interests [8,9,10] led to an exponential increase in the number of publications on this topic afterwards.

Although there is a tendency for a higher interest in the potential benefits of caffeine in women, research analyzing the effect of caffeine on strength in women has been scarce. Most of the studies (68.3%) did not include women in their sample. In contrast, only 12.2% of studies not included men, and only 16.9% (32) of studies show the effect of caffeine on both sexes. Before the 2000s, only 16 women (vs. 110 men) had been included in strength–caffeine studies. Even, only 35 (vs. 248 men) in 2000–2009 and 35 (vs. 385 men) in the 2010–2014 periods were included in investigations on this topic. So, women are under-represented in the literature about caffeine and strength performance. From 1907 to 2014, the presence of women is limited to 9.8%, but even between 2015 and 2022, it is only 22.9% of the total participants. The problem of the under-representation of women is not exclusive to caffeine research, as it has been described in other areas of sports sciences, although with little disparity [53]. Analyzing the three major sports sciences journals, Costello et al. [53] found that only 39% of participants in the studies published with samples of humans were women. Our data showed an even more biased effect and were consistent with previous research in the caffeine context of sports sciences [54]. Salinero et al. [54] analyzed a total of 362 original investigations about the effects of caffeine on physical performance. Their results showed that 703 participants were women from 5321 individuals, which represented only 13.2% of the total sample. In the same line, Grgic et al. [18] in their umbrella review found that in all the included meta-analyses, 72–100% of the pooled sample participants were men, suggesting that more primary studies should be conducted among women to improve the generalizability of the findings. Overall, although current research in other areas of sports sciences reflects that the magnitude of the ergogenic effect of caffeine is similar in men and women when the dose is standardized to body mass [55,56], and women obtain benefit from the caffeine in all phases of their menstrual cycle [57,58,59], more research is necessary to adequately establish the dose–response effect of caffeine on strength performance in active women and female athletes.

Interestingly, until 2015, only four studies employed dose–response designs to establish the association between caffeine amount and the magnitude of its ergogenic benefit on strength. In 1980, Bugyi [60], compared 167, 424 and 500 mg of caffeine, and in 1990 [46], Jacobson and Edwards compared 300 vs. 600 mg of caffeine. It was not until 2012 that the first dose–response study was performed with doses adjusted to the participant’s body mass. Del Coso et al. [61] compared 1 and 3 mg/kg of caffeine, and one year later, Pallarés et al. [62] compared 3, 6, and 9 mg/kg of caffeine, both on exercise protocols of increasing load. Since then, 20 studies have compared doses between 1 and 12 mg/kg. Curiously, only studies with dose–response designs included doses lower than 1.7 mg/kg and higher than 7 mg/kg, suggesting that these dose–response studies are key to understanding the effect of low/high doses of caffeine on strength performance. Even with the presence of these studies, the identification of the dose that produces the highest performance benefit is complex, and it has to be helped by the study of meta-analytic findings. Nowadays, there is a consensus to consider that moderate doses (2–6 mg/kg) of caffeine are necessary to improve strength performance [13,16] with a similar benefit in terms of magnitude within this range. Doses higher than 6 mg/kg are also ergogenic for strength-related variables, but the prevalence of side effects habitually increases along with the dose [13,62,63].

As far as side effects are concerned, only a small proportion of studies about caffeine and strength performance have included the assessment of the frequency or magnitude of typical side effects as study variables (only 50 studies, 26.5%), even in the investigations of the last few years. This aspect is probably of greater interest in investigations associated with health outcomes after caffeine ingestion. However, the study of the benefits and drawbacks of caffeine in the sports context is key to establishing the convenience of caffeine supplementation in athletes. Only one study conducted in the 20th century included the measurement of caffeine-induced side effects as an aim of study, while the interest in side effects associated with caffeine supplementation in the sporting context has become more relevant in the last years. However, it is worth mentioning that systematic reviews had already established the safety of moderate doses of caffeine in sports (3 to 6 mg/kg), at least for healthy and active individuals [63]. The appearance and popularity of caffeinated energy drinks in the market of sports-related foodstuffs is another variable that may have led to this increase in the study of side effects over time, as this type of drinks has been considered harmful in other contexts, especially in younger individuals [64]. The intensive use that some populations have regarding caffeine drinks and foodstuffs (in and out of sport context), leading to the intake of high caffeine doses, may suggest the need to monitor the downsides of caffeine supplementation on the athlete, especially for those using doses > 6 mg/kg.

The study of caffeine properties on strength has included another topic of discrepancy over the years: the use of exercise located in muscles of the upper vs. lower body. Until 2010, only eight (27.6%) studies had employed protocols to assess caffeine’s ergogenicity in the upper body strength, 16 (55.2%) assessed its effects on the lower body and five (17.2%) researchers had studied caffeine effects in both upper and lower body strength performance. In most recent years, this difference has disappeared as the proportion of studies investigating caffeine’s ergogenicity in lower and upper body strength exercises is similar. Interestingly, almost 40% of the most recent studies included measurements of both the upper and lower body, which provides a more adequate context to compare the effect of caffeine on strength in exercises that involve different body parts. This interest in studying the potential differences in response to caffeine between upper and lower body exercise has been likely induced by older meta-analyses [65] that reported that the effect of caffeine in lower-body muscle groups is four- to six-fold greater than in upper-body muscle groups. On the other hand, Grgic et al. [15] have reported through a systematic review and meta-analysis that caffeine significantly improved upper but not lower body strength. Even in this context of different findings in meta-analyses, the current investigation shows a clear trend regarding the increase in investigations that study caffeine effects on strength performance in both upper and lower body exercise, which probably will solve current discrepancies shortly.

This study provides an overview of the scientific protocols used to study caffeine’s effects on strength performance over time. The assessment of the methodological limitations or risk of bias of the investigations included in overview reviews is not usually performed [66], but future studies should analyze how the risk of bias in investigations about caffeine’s properties on strength performance has changed over time. In this context, the current overview review presents some limitations that should be acknowledged. We have limited the search and description of studies’ characteristics to studies on caffeine’s effects on strength performance. Therefore, studies’ attributes on caffeine’s benefits on endurance or aerobic performance have not been analyzed. Future investigations should be carried out to determine how the experimental protocols have evolved to study caffeine’s benefits on endurance-based exercise. We only included human healthy participants (regardless of age). Therefore, this analysis is not representative of people with some disease or injury. Future studies should perform a specific analysis for these types of populations, as it is possible that the methods employed to investigate caffeine properties in terms of caffeine dosage and the reporting of side effects are substantially different when including clinical populations.

## 5. Conclusions

The current review depicts that the interest in developing research on the effect of caffeine on strength exercises has increased in recent years, as per the number of studies published. However, since the 1980s, there have been no relevant changes in the methods used to investigate caffeine’s benefits on strength. Overall, the current overview review indicates that the pattern in the study of caffeine’s effects on strength performance has been carried out with experiments including 11–15 healthy adults, using a single and moderate dose of caffeine adjusted to participants’ body mass (~5 mg/kg) in the form of a capsule, with placebo-controlled experimental design, and analyzing the effect of caffeine on the upper and lower body. With this overview review, it is possible to identify conceptual boundaries, such as the paucity of studies with doses of caffeine below 2 mg/kg or above 9 mg/kg, as well as identify gaps for future research, such as the study of the effects of caffeine on strength in women and older adults along with the identification of frequency and magnitude of caffeine-associated side effects.

## Figures and Tables

**Table 1:** Evolution of the main characteristics of the experimental designs of investigations on caffeine effects on strength performance.

Variables		<1980	1980–1999	2000–2009	2010–2014	2015–2022
***n* papers**		3 (1.6%)	8 (4.2%)	18 (9.5%)	29 (15.3%)	131 (69.3%)
**Sample size**	*n*	2.3 ± 0.6	15.5 ± 10.7	15.7 ± 6.9	16.0 ± 7.8 *	19.7 ± 14.4 *
	*n* Male	2.3 ± 0.6	14.7 ± 7.0	13.8 ± 5.6	13.8 ± 6.7	14.6 ± 13.6
	*n* Female	0	2.3 ± 6.0	1.9 ± 4.4	1.3 ± 3.4	4.6 ± 7.8
**Adjusted dose**	Yes	0 (0%)	5 (62.5%)	14 (77.8%)	20 (69.0%)	96 (73.3%)
	No	3 (100%)	3 (37.5%)	4 (22.2%)	9 (31.0%)	35 (26.7%)
**Dose–response**	Yes	0 (0%)	2 (25.0%)	0 (0%)	2 (6.9%)	20 (15.3%)
	No	3 (100%)	6 (75.0%)	18 (100%)	27 (93.1%)	111 (84.7%)
**Dose**	Adjusted (mg/kg)	–	5.8 ± 0.8	5.2 ± 1.3	4.6 ± 1.5	4.8 ± 1.6
	Absolute (mg)	300	437.9 ± 69.0	180.3 ± 90.6	226.1 ± 95.9	298.3 ± 142.4
**Mix with other** **substances**	Yes	1 (33.3%)	1 (12.5%)	6 (33.3%)	16 (55.2%)	27 (20.6%)
	No	2 (66.7%)	7 (87.5%)	12 (66.7%)	13 (44.8%)	104 (79.4%)
**Relationship with other** **co-ingested substances**	Yes	0 (0%)	0 (0%)	2 (11.1%)	2 (6.9%)	15 (11.5%)
	No	3 (100%)	8 (100%)	16 (88.9%)	27 (93.1%)	116 (88.5%)
**Placebo-controlled design**	Yes	2 (66.7%)	8 (100%)	18 (100%)	28 (96.6%)	130 (99.2%)
	No	1 (33.3%)	0 (0%)	0 (0%)	1 (3.4%)	1 (0.8%)
**Caffeine consumption ** **reported**	Yes	0 (0%)	5 (62.5%)	13 (72.2%)	19 (65.5%)	92 (70.2%)
	No	3 (100%)	3 (37.5%)	5 (27.8%)	10 (34.5%)	39 (29.8%)
**Reported side effects**	Yes	0 (0%)	1 (12.5%)	6 (33.3%)	10 (34.5%)	33 (25.2%)
	No	3 (100%)	7 (87.5%)	12 (66.7%)	19 (65.5%)	98 (74.8%)
**Caffeine form**	Capsule	1 (33.3%)	4 (50.0%)	11 (61.1%)	9 (31.0%)	73 (55.7%)
	Beverage	1 (33.3%)	4 (50.0%)	7 (38.9%)	20 (69.0%)	46 (35.1%)
	Gum	0 (0%)	0 (0%)	0 (0%)	0 (0%)	3 (2.3%)
	Mouth rinse	0 (0%)	0 (0%)	0 (0%)	0 (0%)	5 (3.8%)
	Gel	0 (0%)	0 (0%)	0 (0%)	0 (0%)	1 (0.8%)
	Various forms	0 (0%)	0 (0%)	0 (0%)	0 (0%)	1 (0.8%)
	N/A	1 (33.3%)	0 (0%)	0 (0%)	0 (0%)	2 (1.5%)
**Exercise test**	Upper	2 (66.7%)	1 (12.5%)	5 (27.8%)	8 (27.6%)	31 (23.7%)
	Lower	1 (33.3%)	7 (87.5%)	8 (44.4%)	7 (24.1%)	48 (36.6%)
	Both	0 (0%)	0 (0%)	5 (27.8%)	14 (48.3%)	52 (39.7%)

Data indicate the number of studies that fulfilled (or did not) each methodological criterion, with percentages between parentheses. * indicates a significant differences with data <1980 (*p* < 0.05).

## Supplementary Materials

The following supporting information can be downloaded at: https://www.mdpi.com/article/10.3390/nu15051178/s1, Supplementary 1: Full search criteria for databases; and Supplementary 2: Predefined coding spreadsheet of studies included in the review.
